# Adiposity influences airway wall thickness and the asthma phenotype of HIV-associated obstructive lung disease: a cross-sectional study

**DOI:** 10.1186/s12890-016-0274-5

**Published:** 2016-08-04

**Authors:** Julia H. Barton, Alex Ireland, Meghan Fitzpatrick, Cathy Kessinger, Danielle Camp, Renee Weinman, Deborah McMahon, Joseph K. Leader, Fernando Holguin, Sally E. Wenzel, Alison Morris, Matthew R. Gingo

**Affiliations:** 1 Department of Medicine, University of Pittsburgh, Pittsburgh, USA; 2 Department of Immunology, University of Pittsburgh, Pittsburgh, USA; 3 Department of Pediatrics, University of Pittsburgh, Pittsburgh, USA; 4 Division of Pulmonary, Allergy, and Critical Care Medicine, Department of Medicine, University of Pittsburgh, 3459 Fifth Avenue, 628 NW, Pittsburgh, PA 15213 USA

**Keywords:** HIV, Asthma, COPD, Obstructive lung disease, Obesity, Lipodystrophy, Adiponectin

## Abstract

**Background:**

Airflow obstruction, which encompasses several phenotypes, is common among HIV-infected individuals. Obesity and adipose-related inflammation are associated with both COPD (fixed airflow obstruction) and asthma (reversible airflow obstruction) in HIV-uninfected persons, but the relationship to airway inflammation and airflow obstruction in HIV-infected persons is unknown. The objective of this study was to determine if adiposity and adipose-associated inflammation are associated with airway obstruction phenotypes in HIV-infected persons.

**Methods:**

We performed a cross-sectional analysis of 121 HIV-infected individuals assessed with pulmonary function testing, chest CT scans for measures of airway wall thickness (wall area percent [WA%]) and adipose tissue volumes (mediastinal and subcutaneous), as well as HIV- and adipose-related inflammatory markers. Participants were defined as COPD phenotype (post-bronchodilator FEV_1_/FVC < lower limit of normal) or asthma phenotype (doctor-diagnosed asthma or bronchodilator response). Pearson correlation coefficients were calculated between adipose measurements, WA%, and pulmonary function. Multivariable logistic and linear regression models were used to determine associations of airflow obstruction and airway remodeling (WA%) with adipose measurements and participant characteristics.

**Results:**

Twenty-three (19 %) participants were classified as the COPD phenotype and 33 (27 %) were classified as the asthma phenotype. Body mass index (BMI) was similar between those with and without COPD, but higher in those with asthma compared to those without (mean [SD] 30.7 kg/m^2^ [8.1] vs. 26.5 kg/m^2^ [5.3], *p* = 0.008). WA% correlated with greater BMI (*r* = 0.55, *p* < 0.001) and volume of adipose tissue (subcutaneous, *r* = 0.40; *p* < 0.001; mediastinal, *r* = 0.25; *p* = 0.005). Multivariable regression found the COPD phenotype associated with greater age and pack-years smoking; the asthma phenotype with younger age, female gender, smoking history, and lower adiponectin levels; and greater WA% with greater BMI, younger age, higher soluble CD163, and higher CD4 counts.

**Conclusions:**

Adiposity and adipose-related inflammation are associated with an asthma phenotype, but not a COPD phenotype, of obstructive lung disease in HIV-infected persons. Airway wall thickness is associated with adiposity and inflammation. Adipose-related inflammation may play a role in HIV-associated asthma.

## Background

Obstructive lung disease, encompassing many phenotypes of both fixed and reversible airflow obstruction, is common in HIV-infected persons [1–6]. Chronic obstructive pulmonary disease (COPD) in the HIV-infected population is accelerated in smokers and those with poor control of the viral load [1, 3, 7, 8]. Asthma is another commonly diagnosed chronic pulmonary disease in HIV-infected persons [9, 10]. Despite the prevalence of COPD and asthma in HIV-infected persons, little is known about their pathogenesis in this population.

Obesity influences the development of both COPD and asthma in the HIV-uninfected population. Obesity is more prevalent in individuals with mild COPD compared to the general population, however, the causal nature of the relationship between obesity and COPD is unclear [11, 12]. Obesity, central adiposity, and aspects of the metabolic syndrome have been implicated in the pathogenesis of the adult-onset phenotype of asthma [13–17]. Inflammation related to visceral adipose tissue is thought to drive this association [18]. We have previously shown that doctor-diagnosed asthma in HIV is frequently adult-onset, associated with inflammatory markers common in chronic HIV infection, and 2.5 times more likely in obese compared to normal weight HIV-infected persons [9].

Metabolic effects of HIV and highly-active antiretroviral therapy (HAART) that lead to central adiposity and alterations in inflammation may be relevant to the pathogenesis of airway obstruction in HIV [19, 20]. Long-term HIV infection is associated with chronic inflammation and macrophage activation, measured by high-sensitivity C-reactive protein (CRP) and soluble CD163 (sCD163), respectively [21–26]. Increased central adiposity is associated with the alteration of systemic adipokine profiles, including higher leptin (a pro-inflammatory cytokine) and lower adiponectin (an anti-inflammatory cytokine). Adiponectin is lower in HIV-infected persons and in chronic inflammation [27]; and reduced levels of adiponectin have been implicated in several HIV-associated co-morbidities such as cardiovascular disease and neurocognitive dysfunction [28–30]. In the HIV-uninfected population, levels of adiponectin are lower in asthma and paradoxically higher in COPD [31]. The relationship of adiponectin in HIV-associated obstructive lung disease is unknown.

Obstructive lung disease can manifest subjectively as pulmonary symptoms or objectively as pulmonary function changes or airway remodeling detectable on computed tomography (CT) scan [32–34]. Asthma is often diagnosed by doctors based on episodic, recurrent pulmonary symptoms, such as wheezing. Pulmonary symptoms are more common in HIV-infected individuals with doctor-diagnosed asthma than those without asthma [9]. Airway wall thickening correlates with asthma severity, airflow obstruction, and histopathological changes related to asthma [35–37]. Airway remodeling quantitatively measured by CT scan has not been assessed in HIV-infected persons.

In this study, our primary objective was to determine the relationship of adiposity and adipose-related inflammation with obstructive lung disease phenotypes in HIV-infected persons. Our secondary objective was to determine the relationship of adiposity and its associated inflammation with airway remodeling, as measured by airway wall thickness on CT imaging, in HIV-infected persons. We hypothesized that visceral (mediastinal) adipose tissue and adipose-associated inflammatory markers (adiponectin and IL-6) would be associated with airway remodeling and the asthma phenotype of airflow obstruction and no association with the COPD phenotype of airflow obstruction, in HIV-infected persons.

## Methods

### Participants

This study was a cross-sectional secondary analysis of individuals with documented HIV infection who were 18 years of age or older, recruited between July 1, 2007 and April 30, 2010 from the HIV/AIDS clinic at the University of Pittsburgh Medical Center using posted advertisements and by contacting patients in a research registry. Exclusion criteria included any contraindication to pulmonary function testing and those with increased respiratory symptoms or fevers within the prior 4 weeks to exclude active pulmonary infection. Participants who completed a CT scan of the chest in the established cohort were included in this study. A description of the group and pulmonary function data from the primary analysis has been published previously [2, 9]. Participants signed written informed consent, and the University of Pittsburgh IRB approved the protocol.

### Data collection

Demographic and clinical data, including age, gender, race, smoking status, and illicit drug use were collected through standardized interviews by research coordinators, and if unable to obtain, were then collected through a review of the medical record. A modified version of the American Thoracic Society (ATS) Division of Lung Diseases questionnaire was used to collect information on respiratory symptoms [38]. Participants completed the questionnaire on the same day as blood collection, pulmonary function testing, and CT imaging. A prior diagnosis of asthma was ascertained by asking participants if their doctor had ever told them they had asthma (doctor-diagnosed asthma), in addition to several other pulmonary-related diseases such as COPD or sarcoidosis [9].

### Metabolic, immunologic and serologic parameters

Height and weight were measured to calculate BMI. Induced sputum samples were collected for sputum cell counts [39]. Peripheral blood samples were collected at the time of enrollment, and serum or plasma aliquots were stored at -80C ranging from 2 to 6 years and assayed following a single thaw. Markers of inflammation (interleukin [IL-6], high-sensitivity C-reactive protein [CRP]), monocyte/macrophage activation (sCD163) and the adipokine adiponectin were assayed using ELISA (R&D Systems; Minneapolis, MN). Total IgE was also measured in the clinical laboratory to assess for an association between allergic/atopic measures and prevalent airway obstruction phenotypes. Leptin was not measured because blood samples were obtained in a non-fasting state. The CD4^+^ T-lymphocyte cell count and the plasma HIV ribonucleic acid (RNA) level within the prior three months were obtained from medical record review.

### Pulmonary function testing

All participants completed pre- and post-bronchodilator spirometry (480 μg of albuterol administered through a spacer from a metered-dose inhaler) and single breath diffusing capacity for carbon monoxide (DL_CO_) in accordance with ATS standards [40, 41]. The percent predicted forced expiratory volume in 1 second (FEV_1_) and forced expiratory capacity (FVC) were calculated using the Hankinson prediction equations [42]. DL_CO_ percent predicted was determined using equations from Neas et al and corrected for hemoglobin and carboxyhemoglobin [43].

### Imaging parameters

CT of the chest was used to determine airway wall thickness (WA%) in the smallest one-third of measurable airways. We used a fully automated computer scheme to detect and quantify airway sections depicted in axial section of the CT examination as previously described [44].

Mediastinal (a surrogate for visceral adipose tissue in the chest) and subcutaneous adipose volumes were determined using commercially available software (sliceOmatic, Tomovision; Magog, Canada). The non-contrast chest CT scans, collected as part of the parent study [45], were processed with a standard algorithm in 2.5 mm thickness. Adipose tissue was defined as voxels in Hu range of -190 to -30 [46]. The subcutaneous region included this Hu range extending from skin to fascia circumferentially. The borders of the mediastinum were defined laterally by the edge of the most lateral mediastinal structure (superior vena cava, right atrium, left ventricle, descending aorta, and main pulmonary artery), anteriorly by the sternum, and posteriorly by the vertebral body or the descending aorta, whichever was the more anterior structure. The superior margin of the mediastinum was defined by the first slice inferior to the aortic arch, and the inferior margin was defined by the most inferior slice where the diaphragm does not touch the heart.

To determine the inter-reader reliability of adipose measures, adipose tissue measurements from a subset of CT scans [10] were measured independently by two different investigators (JHB, MRG), blinded to patient characteristics.

### Statistical analysis

Participants were classified as having a COPD phenotype of airflow obstruction (post-bronchodilator FEV_1_/FVC less than the lower limit of normal – below the 95 % confidence interval) or an asthma phenotype of airflow obstruction (either doctor-diagnosed asthma or a bronchodilator response on pulmonary function testing in accordance with ATS standards) [47]. We used this composite of doctor-diagnosed asthma or bronchodilator responsiveness because the pulmonary function testing was done for research purposes and did not include provocation testing, so participants with treated and well-controlled asthma may not have had airflow obstruction or reversibility at the time of the study visit. Participants with overlapping definitions of airflow obstruction were included in both the COPD and asthma groups for analysis.

Participant characteristics and measures were summarized for all participants and compared between those with and without the COPD phenotype or the asthma phenotype of airflow obstruction. Data were normalized using logarithmic or square root transformation if necessary. Parametric testing was performed with t-test, and ranksum test was used for non-parametric comparisons. Categorical and dichotomous variables were compared between those with and without the COPD phenotype or the asthma phenotype of airflow obstruction using chi-square test. Pearson correlation coefficients were used to determine correlations between WA% and lung function. To determine inter-reader reliability of CT adipose measures, Lin’s concordance correlation coefficients were calculated for both subcutaneous and mediastinal adipose tissue measures. Multivariable logistic regression was performed to determine if participant characteristics (age, gender, race, BMI, smoking status, viral load), adipose measures (subcutaneous and visceral adipose volumes), and inflammatory markers (CRP, adiponectin, IgE) were independently associated with the COPD phenotype or the asthma phenotype of airflow obstruction. Linear regression was used to determine associations between airflow obstruction and WA% with the same series of variables used in the logistic regression including participant characteristics, CT adipose measures, and inflammatory markers. Stepwise regression was used for both linear and logistic model selection, including variables significant at a level of *p* < 0.1 in univariate analyses. Logistic models were additionally tested for fit with Hosmer-Lemeshow goodness-of-fit test [48]. Statistical analyses were conducted using StataSE version 13 (StataCorp LP, College Station, TX).

## Results

A total of 121 HIV-infected participants were evaluated. Overall, the mean (SD) age of all participants was 45.1 (9.8) years, and the majority were men (67.8 %) with a history of smoking (82.6 %) (Table 1). Most participants were on antiretroviral therapy (ART) (87.6 %) with a mean CD4 count of 599.1 cells/μL, and 84 (69.5 %) had an HIV RNA viral level <50 copies/mL. Twenty-three (19 %) participants had the COPD phenotype of airflow obstruction. Thirty-three (27 %) participants were classified as having the asthma phenotype of airflow obstruction; 25 of them had doctor-diagnosed asthma, six of whom also had a bronchodilator response, and eight participants had a bronchodilator response without prior doctor-diagnosed asthma. Twelve (10 %) participants fit both definitions of the COPD and asthma phenotypes, and were included in both groups for analysis.

**Table 1:** Participant characteristics by phenotype of airflow obstruction

	COPD (*n* = 23)	No COPD (*n* = 98)	*p*-value	Asthma^a^ (*n* = 33)	No asthma (*n* = 88)	*p*-value
Age, mean (SD)	50.4 (5.7)	43.9 (10.1)	<0.001	43.0 (9.5)	46.0 (9.8)	0.14
Female, n (%)	6 (26.1)	33 (33.7)	0.48	15 (45.5)	24 (27.3)	0.06
African American, n (%)	13 (56.5)	53 (54.1)	0.83	21 (63.6)	43 (48.9)	0.43
BMI (kg/m^2^), mean (SD)	27.0 (7.9)	27.8 (6.1)	0.58	30.7 (8.1)	26.5 (5.3)	0.008
Smoke status, n (%)			0.18			0.13
Never	1 (4.4)	20 (20.4)		2 (6.1)	19 (21.6)	
Former	7 (30.4)	24 (24.5)		21 (23.9)	10 (30.3)	
Current	15 (65.2)	54 (55.1)		21 (63.6)	48 (54.6)	
Pack-years smoked, median (range)	20.0 (0-102)	9.8 (0-45)	<0.001	13.5 (0-102)	10.8 (0-75)	0.33
Intravenous drug use (ever), n (%)	3 (13.0)	3 (3.1)	0.05	1 (3.0)	5 (5.7)	0.99
Cocaine use (ever), n (%)	5 (21.7)	23 (23.5)	0.86	5 (15.2)	23 (26.1)	0.20
Marijuana use (ever), n (%)	9 (39.1)	56 (57.1)	0.12	15 (45.5)	50 (56.8)	0.26
HAART use, n (%)	21 (91.3)	85 (86.7)	0.55	28 (84.9)	78 (88.6)	0.57
CD4 count (cells/μl), mean (SD)	578.6 (270.4)	603.5 (339.6)	0.75	596.7 (299.1)	599.9 (338.6)	0.96
HIV RNA level <50copies/mL, n (%)	16 (69.6)	68 (69.4)	0.99	19 (57.6)	65 (73.9)	0.08
Doctor-diagnosed asthma	8 (34.8)	17 (17.4)	0.99	25 (75.8)	0	na
Bronchodilator response	6 (26.1)	8 (8.2)	0.02	14 (42.4)	0	na
Cough	9 (39.1)	26 (26.5)	0.23	14 (42.4)	21 (23.9)	0.05
Phlegm	9 (39.1)	38 (38.8)	0.98	14 (42.4)	33 (37.5)	0.62
Wheeze	12 (52.2)	31 (31.6)	0.06	19 (57.6)	24 (27.3)	0.002
Dyspnea	10 (43.5)	35 (35.7)	0.49	14 (42.4)	31 (35.2)	0.47
pre-BD FEV_1_ %pred, mean (SD)	72.6 (19.0)	95.8 (16.7)	<0.001	77.7 (20.6)	96.5 (16.2)	<0.001
pre-BD FEV_1_/FVC, mean (SD)	0.61 (0.09)	0.78 (0.06)	<0.001	0.69 (0.12)	0.77 (0.08)	<0.001
post-BD FEV_1_/FVC, mean (SD)	0.62 (0.08)	0.81 (0.06)	<0.001	0.73 (0.12)	0.80 (0.08)	0.003
post-BD FEV_1_/FVC < LLN, n (%)	23 (100)	0 (0)	na	12 (36.4)	11 (12.5)	0.003
DLco % predicted, mean (SD)	0.57 (0.15)	0.68 (0.13)	<0.001	0.63 (0.13)	0.67 (0.14)	0.13

Abbreviations: *SD* Standard deviation, *BMI* Body Mass Index, *HAART* Highly active antiretroviral therapy, *BD* Bronchodilator, *FEV1* Forced expiratory volume in 1 second, *FVC* Forced vital capacity, *%pred* Percent predicted, *LLN* Lower limit of normal, *DLco* Diffusion capacity of the lung for carbon monoxide

^a^Asthma phenotype is defined by a history of doctor-diagnosed asthma or a bronchodilator response during pulmonary function testing (Increase in FEV_1_ or FVC of greater than 200 ml and 12 %)

**Fig. 1:**
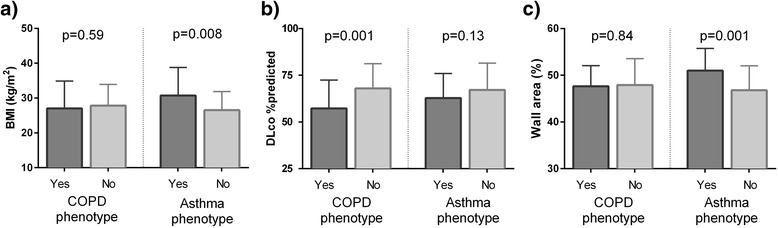
Mean and standard deviation (error bar) of body mass index (BMI) (**a**), diffusing capacity of carbon monoxide (DL_CO_) % predicted (**b**), and wall area % (**c**) in those with and without a COPD phenotype of airflow obstruction (post-bronchodilator forced expiratory volume at 1 second/forced vital capacity less than the lower limit of normal) and in those with and without an asthma phenotype of airflow obstruction (doctor-diagnosed asthma or bronchodilator response)

Participant characteristics differed between those with and without the COPD phenotype of airflow obstruction. Those with the COPD phenotype were older, had heavier smoking history as measured by pack-years, and greater intravenous drug use (Table 1). BMI did not differ between those with and without the COPD phenotype of airflow obstruction (mean [SD] 27.0 kg/m^2^ [7.9] vs. 27.8 kg/m^2^ [6.1], *p* = 0.59) (Fig. 1). Participant demographics were similar between those with and without the asthma phenotype of airflow obstruction, except for BMI (mean [SD] 30.7 kg/m^2^ [8.1] vs. 26.5 kg/m^2^ [5.3], *p* = 0.008), and female sex (n, 15 vs. 24, *p* = 0.06).

Those with the COPD phenotype had lower pre- and post-bronchodilator FEV_1_ %predicted and FEV_1_/FVC as expected by definition, compared to those without COPD. The participants with the asthma phenotype of obstruction had lower pre-BD FEV_1_ (78 %[21] vs. 97 %[16]; *p* = <0.001) and pre-BD FEV_1_/FVC (69 %[12] vs. 77 %[8]; *p* = <0.001) compared to those without asthma. DL_CO_ was moderately reduced amongst all participants (mean, 66 %); however, it was lower in participants with the COPD phenotype compared to those without COPD (57 %[15] vs. 68 %[13], *p* = <0.001). DL_CO_ was no different in those with the asthma phenotype of obstruction compared to those without asthma (63 %[13] vs. 67 %[14], *p* = 0.13) (Fig. 1).

Several inflammatory markers were different between the two phenotypes of airflow obstruction (Table 2). CRP levels were higher in participants with both phenotypes of airflow obstruction (COPD *p* = 0.04, asthma *p* = 0.04) versus those without airflow obstruction. The total IgE level was elevated in the asthma phenotype group of airflow obstruction (*p* = 0.03); however, there was no difference in the number of participants with elevated sputum eosinophils. Adiponectin tended to be lower in the asthma phenotype group compared to those without asthma (*p* = 0.07). There was no difference in the level of sCD163 amongst all participants. There was no difference in the level of IL-6 between those with or without COPD, however, those with the asthma phenotype of obstruction had higher IL-6 levels compared to those without asthma (*p* = 0.03).

**Table 2:** Inflammatory markers and CT measurements by phenotype of airflow obstruction

	COPD (*n* = 23)	No COPD (*n* = 98)	*p*-value	Asthma^a^ (*n* = 33)	No asthma (*n* = 88)	*p*-value
Sputum eosinophils >1.53 %, (*n* = 108)^b^	3 (13.6)	8 (9.3)	0.55	5 (16.1)	6 (7.8)	0.20
Sputum neutrophils (%), mean (SD)	55.9 (19.6)	50.6 (19.6)	0.27	50.0 (22.4)	52.4 (18.5)	0.57
C-reactive protein (mg/L), median (range) (*n* = 117)	3.4 (0-74.2)	1.1 (0-107.6)	0.04	2.3 (0-77.6)	1.1 (0-107.6)	0.04
IgE level (IU/mL) median (range) (*n* = 116)	33.8 (2.2-1359.0)	41.5 (0.8-2758.5)	0.60	83.9 (5.1-1447.0)	28.7 (0.8-2758.5)	0.03
Soluble CD163 (ng/mL), median (range) (*n* = 103)	887.9 (381.5-1999.3)	670.0 (180.1-2381.5)	0.22	726.9 (324.1-2193.1)	682.5 (180.1-2381.5)	0.11
IL-6 > median, n (%) (*n* = 98)	11 (64.7)	38 (46.9)	0.18	19 (67.9)	30 (42.9)	0.03
Adiponectin (ng/mL), median (range) (*n* = 115)	5038 (0-14590)	2822 (0-22611)	0.10	2195 (0-12663)	3713 (0-22611)	0.07
Wall area % (*n* = 117), mean (SD)	47.6 (4.4)	47.9 (5.7)	0.84	51.0 (4.7)	46.8 (5.3)	<0.001
SubQ adipose volume (cm^3^), median (range)^c^	22.9 (3.4-66.8)	26.8 (2.9-85.1)	0.30	33.3 (4.0-85.1)	22.1 (2.9-77.7)	0.004
Mediastinal adipose volume (cm^3^), median (range)^c^	3.3 (1.8-13.9)	4.0 (1.1-13.5)	0.40	3.9 (1.4-13.9)	4.0 (1.1-11.6)	0.33
Mediastinal/SubQ, median (range)	0.19 (0.08-0.63)	0.17 (0.04-0.62)	0.47	0.15 (0.06-0.58)	0.13 (0.04-0.63)	0.02

Abbreviations: *SD* Standard deviation, *IQR* interquartile range, *IL* Interleukin, *IFN* Interferon, *SubQ* Subcutaneous

^a^Asthma phenotype is defined by a history of doctor-diagnosed asthma or a bronchodilator response during pulmonary function testing

^b^Sputum eosinophil count of 1.53 % defined as the upper limit of normal

^c^Adipose volume is standardized by dividing per 5 mm measured in the z-axis

There was excellent inter-reader reliability for subcutaneous (*r* = 0.955; *p* < 0.001) and mediastinal (*r* = 0.995; *p* < 0.001) adipose measures. There was no difference in the volumes of adipose tissue in the mediastinum or the subcutaneous regions of those with and without the COPD phenotype of obstruction (Table 2). Participants with the asthma phenotype of obstruction had a greater volume of subcutaneous adipose tissue (33.3 cm^3^ vs. 22.1 cm^3^, *p* = 0.004), but no difference in the volume of mediastinal adipose tissue compared to those without asthma (3.9 cm^3^ vs. 4.0 cm^3^, *p* = 0.33).

BMI and CT adipose measurements correlated with several markers of inflammation. Adiponectin correlated inversely with BMI (*r* = -0.23, *p* = 0.01), and more strongly with mediastinal adipose tissue (*r* = -0.41, *p* < 0.001) than subcutaneous tissue (*r* = -0.19, *p* = 0.04)(data not shown). Mediastinal adipose tissue correlated inversely with IgE (*r* = -0.19, *p* = 0.04)(data not shown). Both BMI (mean [SD], 30.1 kg/m^2^ [8.2] vs. 25.7 kg/m^2^ [4.0]; *p* = 0.001) and subcutaneous adipose tissue volume (mean (SD) 17.4√cm^3^/slice (5.6) vs. 14.6√cm^3^/slice (4.8); *p* = 0.008) were greater with high IL-6 (data not shown). There was no correlation between adipose tissue and CRP or sCD163 (data not shown).

**Fig. 2:**
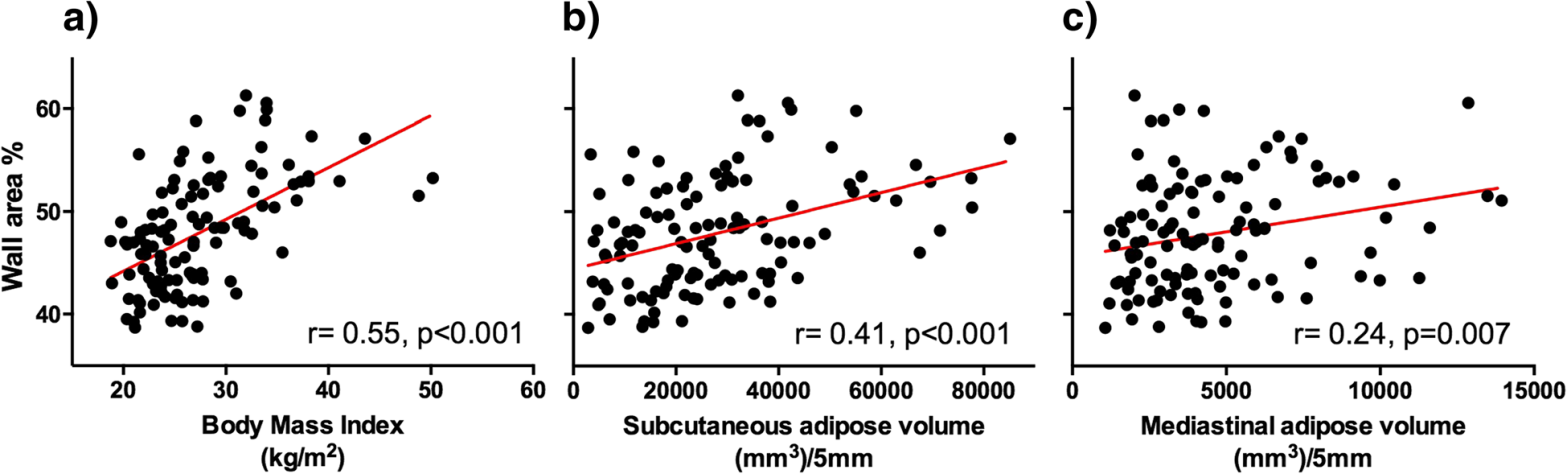
Wall area thickness correlates with measures of adiposity including body mass index (BMI) (**a**), and the volumes of subcutaneous (**b**) and mediastinal (**c**) adipose tissue in a group of HIV-infected persons

Airway wall thickness (WA%) did not differ between those with and without the COPD phenotype of obstruction, but was greater in the group with the asthma phenotype of airflow obstruction (51.0 % vs. 46.8 %; *p* < 0.001) (Fig. 1). Greater WA% was associated with lower pre-BD FEV_1_ (*r* = -0.19; *p* = 0.04); there was no association between WA% and pre-BD FEV_1_/FVC ratio or DL_CO_ (data not shown). Greater WA% was associated with BMI, as well as both subcutaneous and mediastinal adipose volumes (Fig. 2). Greater WA% was associated with symptoms of wheezing (*p* = 0.02) and tended to be associated with phlegm production, but was not associated with cough or dyspnea (data not shown).

We performed multivariable regression models to identify factors associated with the COPD phenotype of airflow obstruction, the asthma phenotype of obstruction, and WA% (Table 3). A list of all variables included in the models can be found in the Methods section. The COPD phenotype was associated with older age and longer smoking history. The asthma phenotype was associated with the female sex, younger age, smoking history, and lower adiponectin values. Greater WA% was associated with greater BMI, younger age, higher sCD163, and higher CD4 counts. People who smoked cigarettes or used cocaine had lower WA%.

**Table 3:** Multivariable analysis models of pulmonary function abnormalities and airway wall thickness with participant characteristics and inflammatory markers

COPD phenotype	OR (95 % CI)	*p*-value
Pack-years smoked, per pack-year	1.05 (1.02-1.08)	*p* = 0.003
Age, per year	1.07 (1.01-1.14)	*p* = 0.03
Asthma phenotype	OR (95 % CI)	*p*-value
Female vs. male	5.48 (1.78-16.9)	*p* = 0.003
Age, per year	0.95 (0.91-0.99)	*p* = 0.04
Smoking history, per pack-years	1.03 (1.00-1.06)	*p* = 0.04
Adiponectin, per ln(ng/mL)	0.98 (0.96-0.99)	*p* = 0.01
Airway wall thickness	Coef. (95 % CI)	*p*-value
Body mass index, per kg/m^2^	0.39 (0.23-0.54)	*p* < 0.001
Age, per year	-0.13 (-0.22-0.05)	*p* = 0.003
Former smoker vs. never smoker	-3.76 (-6.34-1.18)	*p* = 0.005
Current smoker vs. never smoker	-3.21 (-5.57-0.86)	*p* = 0.008
Ever used cocaine vs. never used cocaine	-2.35 (-4.60-0.10)	*p* = 0.04
soluble CD163, per ln (ng/mL)	2.95 (1.26-4.64)	*p* = 0.001
CD4+ T-cells, per 100 cell/μL	0.27 (0.01-0.52)	*p* = 0.04

Abbreviations: *OR* Odds ratio, *Coef* Coefficient, *CI* Confidence interval, *CRP* C-reactive protein, *FEV*_1_ Forced expiratory volume in 1 second, *FVC* Forced vital capacity, *ln* natural logarithm

## Discussion

This study demonstrates that there are unique associations that may be contributing to the pathogenesis of two airway obstruction phenotypes in HIV-infected persons. We found that the COPD phenotype of airflow obstruction was associated with heavier smoking and older age. In contrast, the asthma phenotype of airflow obstruction was associated with the female sex, younger age, smoking history, and lower adiponectin levels. These findings suggest that, while there is some degree of overlap in the two phenotypes, the association of adiponectin, an important adipose-related mediator of inflammation, with the asthma phenotype of obstruction may be mechanistically important in HIV-associated asthma.

Asthma has several phenotypes of disease in the HIV-uninfected population and may, similarly, have several phenotypes in the HIV-infected population. Similar to the general population, there is strong evidence that the allergic phenotype of asthma is present, with elevated total IgE. Additionally, a subset of asthma in HIV may represent the adult-onset, obesity-mediated phenotype. We have previously shown an association between asthma and BMI in HIV [9], suggesting that the increasingly recognized adult-onset obesity phenotype might also be more prevalent in the HIV-infected population [49, 50]. Our univariate analyses highlight the significant association of increasing volume of subcutaneous tissue with the asthma phenotype of airflow obstruction more than mediastinal adipose tissue as we had hypothesized. Increased deposition of visceral adipose tissue is more strongly associated with metabolic dysregulation and inflammation in the HIV-uninfected population; therefore, subcutaneous adipose tissue in the HIV-infected population may be playing a more significant role in asthma pathogenesis. This study also demonstrated a strong correlation between BMI and adipose measures, both subcutaneous and mediastinal, with increased airway wall thickness, a marker of airway inflammation and remodeling. In multivariable analysis, there was a significant correlation between adiponectin and the asthma phenotype of obstruction, an adipose-mediated inflammatory marker. Several studies have shown a link between adiponectin and asthma in obese asthmatics compared to non-obese asthmatics, but this association has not previously been reported in HIV [14, 17, 18].

Alternatively, HIV-associated asthma may be related to chronic HIV-associated inflammation. HIV leads to immune activation and chronic inflammation, with elevated levels of IL-6, CRP, and D-Dimer compared to the general population [21–24]. In our study, CRP tended to be associated with greater airway obstruction. We and others have shown that serum IL-6 is associated with adiposity, suggesting that there is increased inflammation related to obesity as well [51]. In our multivariable regression model, airway remodeling was associated with greater BMI, higher CD4 counts, and higher sCD163 levels, suggesting that macrophage activation from chronic immune activation [25, 26] and obesity may both play a role in airway remodeling, but the degree to which they are related is unclear [52, 53].

Inflammation related to lipodystrophy is an important factor causing HIV-associated non-AIDS diseases. Several other HIV co-morbidities, including cardiovascular disease and neurocognitive dysfunction, have been associated with increased adiposity and inflammation [28–30]. Our findings mirror these associations, suggesting that adipose-related factors, chronic inflammation, and macrophage activation are probable contributors to the increased disease burden of certain types of obstructive lung disease in HIV infection.

It is important to note the global reduction in the DL_CO_ and high smoking rate amongst all participants within this cohort of HIV-infected persons. Diffusion impairment is commonly reduced in the HIV-infected population compared with the general population, even in never smokers [3, 5]. A significantly lower DL_CO_ was a feature of the COPD phenotype, but not the asthma phenotype, confirming that the asthma phenotype is a unique phenotype of airflow obstruction within this population. This phenotype may be related to different factors than those that drive diffusion impairment and COPD. Furthermore, airway wall thickness did not correlate with DL_CO_, only with BMI and FEV_1._

This cohort of HIV-infected persons is representative of the HIV population in the post-ART era, with the majority of participants well-controlled on ART with an average CD4 count > 500cells/μL and HIV RNA levels <50copies/mL. Although there was a high prevalence of tobacco and drug use in this cohort, which may lead to alterations in some of the measured inflammatory markers, these risk factors did not differ between those with and without airflow obstruction, and are not markedly higher than the U.S. HIV population estimates [54, 55]. Another strength of this study is that we used a variety of methods to assess adiposity and its correlation with asthma, including clinical data, radiographic data, and serum biomarkers.

Our study has limitations that need to be considered. It is cross-sectional, and therefore, it is not possible to attribute cause and effect, particularly between obesity and asthma. However, many studies showing an association between obesity and asthma have been corroborated in longitudinal studies and even an intervention study [56–58]. We defined the asthma phenotype of obstruction as having either doctor-diagnosed asthma or a bronchodilator response with pulmonary function testing, and only 42.4 % of those with doctor-diagnosed asthma had a bronchodilator response. The absence of bronchodilator response could be attributed to treated or well-controlled asthma not manifested on pulmonary function testing at the time of the study. The composite definition of the asthma phenotype may lead to heterogeneity in our study group, but we have found pulmonary function and biomarker similarities in participants fitting this composite definition [9]. Bronchodilator responsiveness is more classically seen in asthma; however, bronchodilator reactivity can be seen with fixed airflow obstruction, and in fact, may represent a sub-phenotype of COPD (asthma-COPD overlap syndrome (ACOS) rather than asthma. Despite our inclusion of the overlap participants in both the COPD and asthma airflow obstruction groups during analysis, there was a striking difference between participant characteristics suggesting that participants with the overlap are a unique phenotype. We measured mediastinal adipose tissue because we had access to chest CT scans done as part of the parent study [45]. Mediastinal adipose tissue volume has been shown in other populations to have similar characteristics to abdominal visceral adipose tissue and to correlate with subclinical cardiovascular disease in HIV [59]; however, this measure may not be an adequate reflection of visceral adiposity. Dedicated abdominal CT scans may be more useful in this assessment, but were not available. Also, we used adiponectin as our adipose-mediated inflammatory marker as these were non-fasting blood samples; however, leptin has been more strongly correlated with obesity-related inflammation and asthma. Mediastinal adipose tissue and adiponectin have been correlated with cardiovascular disease in HIV, supporting the validity in these measures as important markers in adipose-mediated inflammation. Finally, the level of associations that were seen in this study were interesting and novel, albeit overall low to moderate in biological associations between the variables.

## Conclusions

In conclusion, there are different features associated with the COPD phenotype versus the asthma phenotype of obstructive lung disease in HIV-infected persons. Adipose-related factors, chronic inflammation, and macrophage activation are probable contributors to the increased disease burden of obstructive lung disease in HIV infection. These differences point out that there is likely not a single mechanism of airway obstruction in HIV, and a better understanding of the airway disease phenotypes and their pathogenesis is needed to optimize treatment of airflow obstruction in HIV.

## Endnote

Not applicable for this study.

## Abbreviations

ATS, American Thoracic Society; BMI, Body mass index; COPD, Chronic obstructive pulmonary disease; CRP, C-reactive protein; DL_CO_, Diffusing capacity for carbon monoxide; FEV_1_, Forced expiratory volume in 1 second; FVC, Forced vital capacity; HAART, Highly active antiretroviral therapy; HIV, Human immunodeficiency virus; IRB, Institutional review board; RNA, ribonucleic acid; SD, Standard deviation; WA%, Airway wall thickness.
